# Discoveries or doubts: a qualitative study of the transformative potential of portfolio meetings

**DOI:** 10.1007/s10459-024-10387-3

**Published:** 2024-10-31

**Authors:** Jenny McDonald, Sylvia Heeneman, Wendy Hu

**Affiliations:** 1 Translational Health Research Institute, School of Medicine, Western Sydney University, Locked Bag 1797, South Penrith, NSW 2751 Australia; 2 Department of Pathology, Maastricht University, Maastricht, The Netherlands; 3 School of Health Profession Education, Maastricht University, Maastricht, The Netherlands

**Keywords:** Portfolios, Medical education, Transformative learning, Qualitative research, Mentoring, Reflection

## Abstract

To adapt to medical school, students need to change their approaches to learning and study. Transformative learning through critical reflection on disorienting learning experiences supports perspective change to direct new activity. We explored how portfolio meetings support changes in students’ perspectives towards learning and study during the transition to studying medicine. This qualitative mixed methods study explored changes in medical students’ perspectives before and after two portfolio meetings with a mentor during the first year of medicine. Adopting a constructionist approach, we analyzed interview transcripts and written reflections from a diverse sample of students using reflexive thematic analysis. The findings were integrated during analysis. Transformative learning theory was our interpretive lens. Our analysis revealed five themes. Two themes represented students’ initial apprehension about portfolio meetings: The Disclosure Dilemma and A Question of Priorities. The theme “Seeing the Big Picture” described new perspectives from reflection on learning while preparing for meetings. Clarity from Dialogue described changed perspectives to learning and study after meetings. In the theme Dialogue Disappointment, meetings perceived as unhelpful led to persisting doubts about the value of portfolio meetings. Transformative learning was evident when students described new insights into their learning leading to goal-setting and new study strategies. When initial meetings were helpful, doubts about portfolio meetings were dispelled, enhancing student engagement in future meetings. Not all meetings were transformative, highlighting the importance of student and mentor preparation and training. Further research is needed to determine whether early portfolio experiences shape later engagement in clinical contexts.

**Supplementary Information:**

The online version contains supplementary material available at 10.1007/s10459-024-10387-3.

## Introduction

First year medical students need to refine their self-regulated learning (SRL) skills to meet the academic load of medicine (Barbosa et al., 2016; Schei 2018). Part of this adaptation requires a shift in goal orientation (Zimmerman & Moylan, 2009) from exam performance to clinical competency (Hall et al., 2021) for study planning. Studying for clinical competency requires the acquisition of conceptual knowledge and skills that can be applied in clinical settings (Hall et al., 2021). This means a major shift in student perspectives about the purpose, meaning and approach to learning and study. Whereas SRL is a helpful cyclical process of self-assessment and planning with goal setting to improve individual performance (Panadero, 2017; Zimmerman & Moylan, 2009), transformative learning (TL) is also needed to change perspectives or mindsets (Friedman, 2022; Mezirow, 1991). A change in perspective or meaning for a situation changes the purpose and direction for future activity (Friedman, 2022; Mezirow, 1991; Taylor & Hamdy, 2013; Taylor, 2012). To successfully adapt to studying in medicine and before setting goals, medical students need to first understand and appreciate how their study should be relevant and directed toward their future clinical competency.

In medical programs, portfolios with mentoring meetings have been designed to support SRL and reflection skills (Challis, 1999; Dallaghan et al., 2020; Driessen, 2017; Mann et al., 2009; Van Tartwijk & Driessen, 2009). While there is evidence that portfolios foster reflection skills (Abouzeid et al., 2021; Driessen et al., 2005; Tan et al., 2022) and goal setting (Hauer et al., 2018), there is limited evidence that portfolios promote SRL in preclinical settings (Abouzeid et al., 2021; Beckers et al., 2016; Buckley et al., 2010; Driessen et al., 2007; Tan et al., 2022) or clinical settings (van der Gulden et al., 2022). In addition, mentors consistently express more satisfaction with the value of portfolios than do students (Driessen, 2017). Although portfolios are recommended to support adaptation to learning in competency-based programs (Hall et al., 2021), there is limited evidence that portfolios with mentoring support students to understand the purpose for, and best approaches to studying in medicine (Altahawi et al., 2012). Unless portfolios help students to understand how their learning and study is related to future clinical competency it is unlikely portfolios will support SRL. Consequently, students will continue to be doubtful about the role and benefits of portfolios.

Current approaches to meetings to discuss portfolios include a combination of coaching, mentoring, and advising (Dekker et al. 2009) to help students make sense of information about their progress and performance and to set goals for learning plans (Dallaghan et al., 2020; Van Tartwijk & Driessen, 2009). With coaching, feedback on performance is provided; with mentoring, professional development is guided, and with advising study advice is offered (Hammoud et al., 2023). Each approach offers different benefits and approaches are usually combined. The success of portfolio meetings in fostering any new perspectives depends on the portfolio curriculum design (Driessen, 2017; Oudkerk Pool et al., 2020), the style and approach of mentors (Loosveld et al., 2022; Meeuwissen et al., 2019) and the role of the portfolio within programs (Arntfield et al., 2016; Lam, 2023; Schrempf et al., 2022; Snadden & Thomas, 1998; van der Gulden et al., 2023; Van Schaik et al., 2013). For example, tensions can be created during portfolio meetings for students and mentors when high stakes assessment is combined with support (Heeneman & de Grave, 2017). The focus on assessment could disrupt the opportunity to guide students to consider the relevance of their learning for later clinical competence.

Transformative learning (TL) theory (Mezirow, 1991; Taylor, 2012) provides a theoretical framework to explain how portfolio meetings might support changes in student perceptions about the purpose for their learning and approach to studying and why there could be variations in student perceptions about portfolio meetings. TL theory posits that when adult learners are supported to critically examine and reassess their assumptions and beliefs in the face of disorientating experiences, a permanent shift in perspective results, creating a new purpose for future activity (Friedman, 2022; Mezirow, 1991; Taylor & Hamdy, 2013; Taylor, 2012). Critical to this process is the combination of sharing disorienting experiences, critical reflection either alone or through dialogue, and readiness to consider and adopt new perspectives (Mezirow, 2003).

However, transformative learning outcomes of portfolio meetings are contingent on whether the portfolio collection present disorienting experiences for discussion (McDonald 2024), the student is ready to share those challenges and learn (Schnepfleitner & Ferreira, 2021; Taylor, 2012), and the mentor has skills in facilitating dialogue and critical reflection (Mezirow, 2003). Mentors could foster perspective changes and approaches to learning by guiding reflection and offering alternative perspectives. This means that portfolio meetings in the first years of a medical program have the potential to support students in understanding the purpose of studying in medicine as a basis for study planning. Whether portfolio meetings support this pivotal step in study planning has not been explored.

In this longitudinal qualitative study of first-year medical students, we aimed to explore how students’ experience preparing for meetings and portfolio meeting discussions influence students’ perceptions of portfolios, portfolio meetings and studying in medicine. We addressed two research questions:

- How do portfolio meetings change medical students’ perspectives on learning and study in the first year of medical school?
- What are the conditions for portfolio meetings that facilitate the transformation of students’ perspectives on learning and study planning during the first years of medical school?

## Methods

This qualitative mixed methods study (Morse, 2009)underpinned by social constructionism (Harré, 2002), explored changes in students’ perceptions of portfolio meetings over the first 18 months of studying medicine from two types of qualitative data from students: individual interviews and written reflections about the portfolio meetings. Social constructionism recognises that knowledge, meaning, and identity are constructed according to context and shaped through dialogue. (Harré, 2002) Analysis of two types of data provided a richer understanding of the students’ perspectives (Varpio et al., 2017). One type of data, in-depth interviews with individual students conducted by an independent research assistant who was not connected to the medical program, provided detailed and confidential responses. These data were compared with students’ written reflections that form part of the portfolio submission in preparation for discussions with an academic adviser during portfolio meetings. These data were linked through their content as the focus of enquiry was similar. However, the written reflections were expected to offer a more positive or optimistic view of portfolio meetings than the interview-derived data. Integration was achieved during thematic (DeJonckheere et al., 2024) and comparative analysis (Bazeley, 2015, 2018). The integrated findings were interpreted using TL theory (Mezirow, 1991; Taylor, 2012). The creation of a visual display supported and illustrated our interpretation of how TL theory explains changing student perceptions before and after portfolio meetings (Dickinson, 2010).

### Context

Our study setting is the Western Sydney University (WSU) 5-year undergraduate medical program, where the portfolio was designed to promote self-regulated learning and reflective practice. The total student cohort of approximately 700 students is culturally and linguistically diverse and includes international, domestic, and indigenous students. The first two years of teaching are predominantly campus-based lectures and small group teaching of biomedical sciences and research skills, professionalism concepts, and introductory clinical skills. During first year students are assessed through class participation, summative written assignments in research skills and professionalism and written examinations for biomedical sciences. Clinical skills are assessed by Objective Structured Clinical Exams (OSCE).

The portfolio is introduced to students in the first week of the medical program in workshops which introduce the purpose of the portfolio, reflective writing and required format for portfolio submission prior to meetings with academic advisers (‘Advisor’). These learning activities aim to support study planning and reflective practice. Students meet with their allocated Advisor to discuss their portfolio twice in first year, and once in the middle of the second year. During these portfolio meetings, the Advisors coach critical reflection on learning, offer study strategies, guide the refinement of the student’s preprepared goals and learning plans, answer questions, and refer when necessary for academic or psychological support. A mix of coaching, mentoring, and advising is provided. The Advisors are experienced teachers within the program with backgrounds in biomedical science and/or health professions. Advisor briefings occur before the meetings to remind advisors that the purpose for the meetings is to facilitate reflection on learning and to help the student refine their learning plans. Examples of questions to facilitate reflection and guidelines for assessment and referral are provided. Students usually meet with the same Advisor for both meetings in first year. Advisors are briefed immediately before they meet with students to remind them of the purpose and format of the portfolio meetings.

In preparation for these portfolio meetings, students submit a presentation using MyKnowledgeMap e-portfolio software (https://www.myknowledgemap.com/). Students are instructed to include in their portfolio presentation two artifacts representing significant learning experiences for each of the four curriculum domains (patient care, health in the community, personal and professional development, and scientific basis of medicine), and a future learning plan. Significant learning experiences are defined as experiences that have helped the student improve or master knowledge or a skill or have taught something memorable, such as how to solve a problem or by showing them what they have learned and are yet to learn. Artifacts may include photo images, videos, certificates of achievement and other assessment tasks. For each artifact, students are asked to write a short reflection explaining the significance of these experiences to their learning. In a workshop to prepare students for their meetings examples of artifact reflections are provided and explained. After the first meeting, subsequent presentation submissions include a written reflection on the impact of the preceding meeting. These longitudinal reflections formed the second of our data sources. During the 30-minute portfolio meeting with Advisors, students present and discuss their chosen artifacts and reflections and a prepared learning plan. These portfolio meetings are compulsory, and the student’s presentation and participation must be satisfactory for progression.

### Sampling and data collection

#### Interview transcripts

This dataset comprised deidentified interview transcripts from 12 interviews with seven students conducted over an 18-month period. When these participants volunteered, they were recruited to participate in a two-year longitudinal study from a first-year cohort of 147 students in March 2020. A research assistant (SR) who did not teach in the medical program conducted semi-structured interviews with the participants over their first two years of study. The participants’ previous study experience and demographic backgrounds varied; three were female, two had no prior higher education, the rest had studied previously in another university course, three were high academic performers, and four lived in English-speaking households. Individual details are not provided to protect anonymity. Part of the interview data, not that used in this study, was used for another study on student adaptation of informed self-assessment skills (McDonald et al. 2022).

A semi-structured interview schedule was developed through a literature review, research team discussion and testing with two medical students, a business studies student, and members of the research team. The interviews were conducted by videoconference between May 2020 and November 2021, with a duration of 60–120 min. These interviews occurred just prior to or after the participants first met with their Advisor (T1), after their second meeting (T2), or after their meeting with their Advisor in the second year (T3). Examples of questions from the T2 interviews are provided in Table 1. The interview schedules and the guidelines for the written reflection are provided in the supplementary material.

#### Written reflections

The second dataset included deidentified written reflections on two portfolio meetings in first year, May 2020 (R1) and September 2020 (R2), extracted from first-year cohorts’ portfolio meeting presentations. For this dataset, a sample of 42 participants, including all the interview participants, was purposefully selected by the SR from the same first-year cohort using demographic data drawn from university records. To ensure maximum variation in student perspectives, the sample included the full range of characteristics known to be associated with academic performance, such as age, gender and ethnicity (Ferguson et al., 2002; Woolf et al., 2011) and past study experience. The participant demographic and academic details were aggregated to preserve privacy and are provided in Table 2.

These written reflections (100–500 words each) were mostly descriptive, providing reasons for including a particular artifact based on the students’ own judgment. Elements of critical reflection, such as consideration of alternative perspectives (Hatton & Smith, 1995), were uncommon. Table 1 presents the guidelines provided to students for their written reflections about the portfolio meetings with examples of corresponding questions from the second interview schedule.

**Fig. 1:**
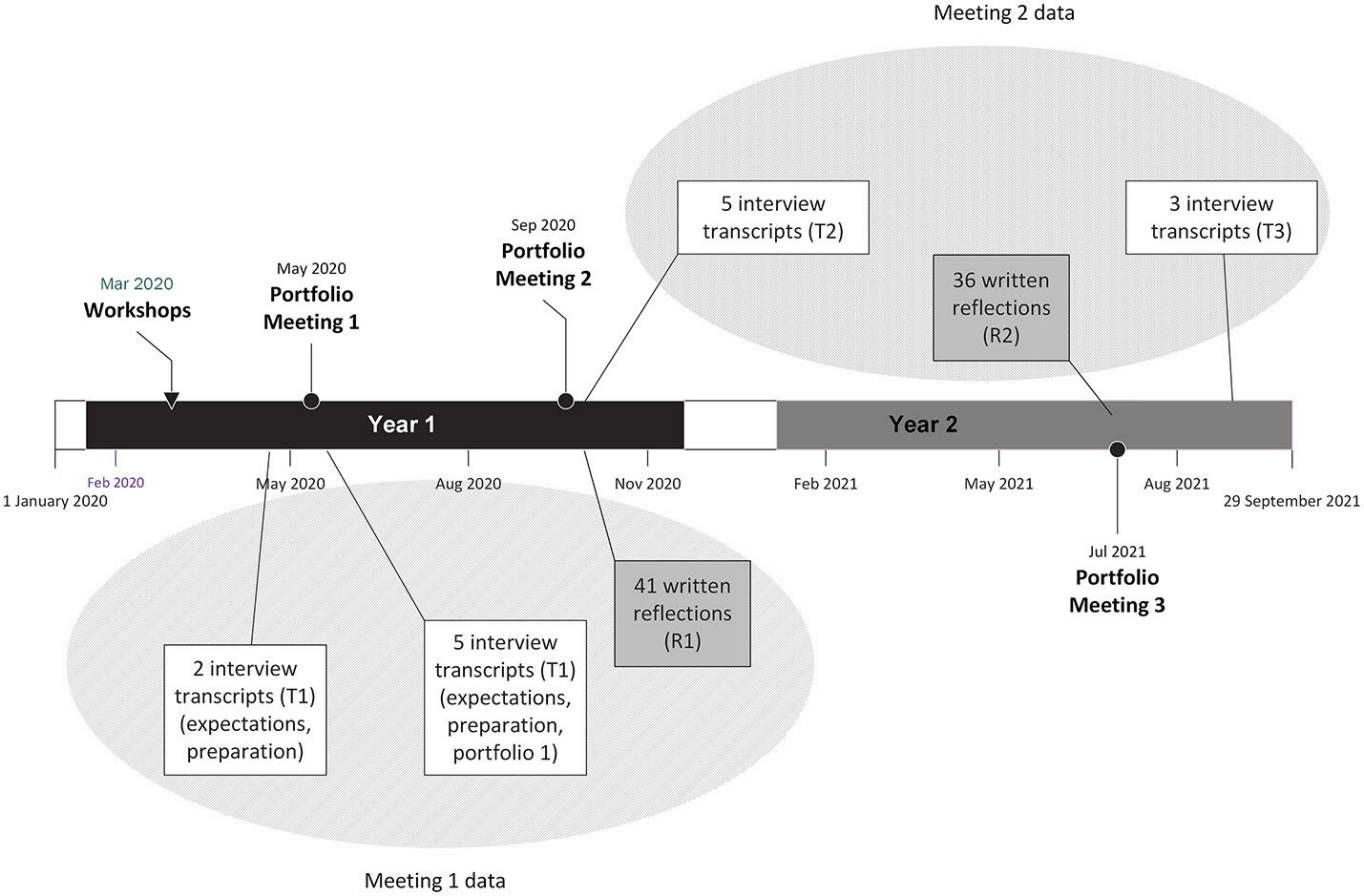
The datasets and study timeline. The figure shows the timing and connections between the data sources. Seven interview transcripts and 41 written reflections related to Portfolio Meeting 1 in May 2020 and 8 interview transcripts and 36 written reflections related to Portfolio Meeting 2 in September 2020

Figure 1 provides an overview of the timeline and two data sources.

**Table 1:** Example questions from semi-structured interview schedule at T2 and written reflection task guidelines

Examples of questions from semi-structured interview schedule (T2)	Guidelines for written reflection about portfolio meetings (R1, R2)
Tell me about your portfolio meeting?	Describe and evaluate the portfolio meeting.
What did you choose to put in your presentation? Why?	What did you learn from the preparation for the portfolio meeting and the experience of the meeting?
What were your thoughts about your achievements over the year when you looked at your showcase?	Did this change your view of your progress?
Did it lead to any changes in your learning?	Have you made any changes to your learning plan since your portfolio meeting?

**Table 2:** Participant demographics and academic performance

Characteristic		*n*		*n*		*n*
Gender	Male	23	Female	19		
Prior study	University	18	High School	24		
Age (years)*	18–20	17	> 20	25		
Residency	Domestic	32	International	10		
Language spoken at home	English	22	Language other than English	20		
Year 1 Performance	High*	22	Pass	17	Fail	3
Year 2 Performance	High*	13	Pass	21	Fail	5

*Age has been dichotomized as median age for cohort or below (18–20 years) and above (> 20 years)

#### Information power

We considered our sample size sufficient in terms of information power (Malterud et al., 2016) to address our research aim to describe a phenomenon drawing on an established theory as our interpretative lens The diversity within both samples and the longitudinal nature of our dataset provided breadth as the reflections provided in each were targeted for different audiences, either the researcher or for Advisors and at different times. The interviews provided rich data and depth as they were at least an hour long and conversational in nature. Open-ended questions allowed the participants to provide elaborated responses and the interviewer explored responses by asking for examples and for further explanation. As all but one of the participants were interviewed more than once, the second or third interview provided opportunity to revisit past discussions to explore change. The two data types therefore provided multiple perspectives and allowed cross-case, cross-source, and temporal comparisons. (LaDonna et al., 2021).

### Data analysis, integration and interpretation

The interviews were audio-recorded and transcribed. All data were deidentified before being uploaded into NVivo v12.0, released 2022 (QRS International Pty Ltd.) and then analyzed. Data were first coded by attribute (participant identifier, data type and the timing of collection in relation to the portfolio meetings). We used the six-phase analytical process of reflexive thematic analysis (Braun & Clarke, 2019; Byrne, 2022).

The deidentified written reflections and interview transcripts were first read and annotated by JM to develop familiarity with the dataset. The data types were then inductively and iteratively coded separately. These initial codes were reviewed, compared, and categorized to create a final set codes for the combined dataset ensuring concepts were applicable to both data types (DeJonckheere et al., 2024). Once coding of the combined dataset was finalized, comparative analysis using a matrix-based approach was used to identify patterns and variations in the participants’ perceptions before and after their portfolio meetings, and within and between the two sources of data. Journalling documented how our analysis developed over time. Regular discussions with the research team helped to enhance our identification and interpretation of patterns in the coded data. Themes were then generated that best described “patterns of shared meanings” (Braun & Clarke, 2019, p. 593; Byrne, 2022) across the dataset and time periods.

**Fig. 2:**
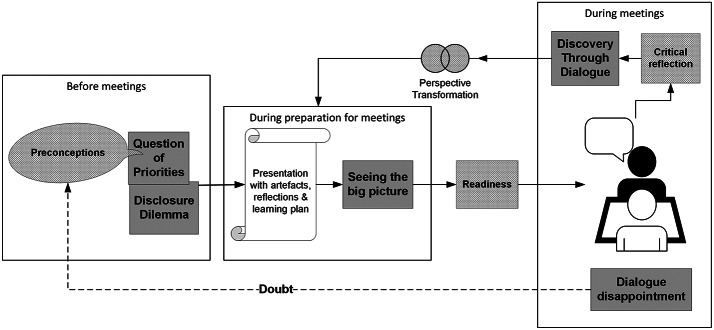
A visual display shows the interconnection between portfolio tasks (white), our themes (gray), and transformative learning processes and outcomes (cross-hatched). Preconceptions influence preparation for meetings. Preparation influences readiness to engage and meeting outcomes. Discovery Through Dialogue supported perspective transformation and future meeting preparation. Dialogue disappointment caused doubt (signified by a broken line) and persistence of preconceptions

For the final stage of analysis, concepts from transformative learning (TL) theory (Mezirow, 1991; Taylor, 2012) were used as sensitizing lenses to explore how our final themes could explain changes in participant descriptions of their portfolio learning experiences before and after their portfolio meetings. It was anticipated that if portfolio meetings support successful study adaptation through TL, there should be evidence that portfolio tasks lead to changes in students’ perspectives about their learning, the purpose for their learning and related future activities. The creation of a visual display supported this interpretation and illustrated how our themes related to portfolio tasks, TL processes and changing student perceptions. See Fig. 2.

### Researchers and reflexivity

Our research team comprised two medical educators from the study site (WSU), both with clinical and teaching experience (JM, WH), and a senior medical educator from the Netherlands (SH). Our interpretations of the patterns in the data arising from participants’ accounts of their portfolio learning were shaped by our own experiences, expectations and assumptions as medical educators. The lead researcher (JM) is the academic lead for the portfolio at WSU, and JM and WH are both portfolio Advisors. This meant that our interpretation of the data was influenced by our familiarity with the WSU program, students, and its cultural context. These ‘insider’ interpretations were shared and compared with SH’s insights as a portfolio researcher and medical educator with extensive experience in another medical program in another country with a different portfolio design.

### Ethics

Ethical approval for this study was granted by the Western Sydney University Human Research Ethics Committee (ID No H9989 AM8385).

## Results

Over the study period, five themes encapsulated the participants’ perceptions of the portfolio meetings before, including during the preparation for, and after the first and second meetings. Two themes described initial reservations and predominated in the data collected before their first meeting: *Disclosure Dilemma* and *A Question of Priorities.* The theme *Seeing the Big Picture* encapsulates participants’ insights from preparing for the meetings when participants curate and reflect on their learning experiences. The theme *Clarity from Dialogue* describes changes in perspectives on learning and study after the first and second meetings. The fifth theme: *Dialogue Disappointment* describes experiences of meetings when perspectives the benefits of portfolio meetings remain unchanged.

In the illustrative quotes below, participants are denoted as P, interview transcript data as T1 (first), T2 (second) or T3 (third) interviews, and written reflection data as R1 (reflection related to first portfolio meeting) or R2 (reflection related to second portfolio meeting).

### Disclosure dilemma

In the interview transcripts, participants explained their preconceptions of portfolio meetings. These were misapprehensions related to the disclosure of difficulties. After the first meeting, P1 explained, *“People don’t like to sit in front of a stranger*, *a lecturer or whatever*, *and talk about things they’ve done badly”* (P1, T1).

For P1, honesty in disclosure underscored ongoing apprehension that the Advisor would not have the right information to give the best advice:

*So*, *inevitably a student’s presentation as “a slightly artificial representation of what you’ve really done because no one’s gonna put in their showcase things that went badly or things they don’t like. They’re only gonna put in things that show them in a positive light and meet the criteria.* (P1, T2)

This misapprehension persisted for some, with P6 saying *“it’s really a bit daunting” to “open up” about yourself.* (P6, T2)

For others, honesty was described as a powerful enabler of new insights after portfolio meetings. Some participants were open to disclosure, even for their first meeting: [the meeting] *pushed me to understand myself better*, *understand where my weaknesses are*, *especially in learning*, *well*, *medicine*, *and how it was something that I needed to improve*, *just in general*, *about my thinking skills”.* (P7, T1)

P21 concurred, in a written reflection:

*What I liked most about the interview* [portfolio meeting] *was the ability to actively voice out my progress in different areas of med school. This combined with the actual creation of the portfolio*, *really opened up how I was doing to myself. It made me a bit more honest with myself*, *made me realize inefficiencies in my methods and ways I can improve*. (P21, R1)

### A question of priorities

Another misapprehension for participants related to perceived lack of benefit from portfolio tasks and meetings. In interview transcripts soon after the first portfolio meeting, participants disclosed how portfolio preparation had seemed a time-wasting exercise in the face of competing study priorities. P7 explained *that “it’s very difficult to move away from the traditional sort of standpoint of studying for*, *well*, *let’s say the scientific part of medicine”* (P7 T1), and P6 was “*not sure” that* the effort would give *“enough benefit”.* (P6, T1) This theme was not evident in the written reflections expecting that reflections would be read by the participant’s Advisor.

### Seeing the big picture

In both written reflections and interviews, participants described how preparing for meetings allowed them to obtain a broader view of their progress and appreciate the value of reflection. Reflections on a range of learning experiences from across the curriculum brought three insights:

Firstly, a sense of direction:

*Preparing my first portfolio showcase allowed me to reflect on what I have accomplished in the first semester of medical school thus far and also begin to realize how far I still need to progress.* (P23, R1)

Secondly, insights about into the integrated curriculum and its relevance to medical practice came from bringing together evidence of their learning presentation for a presentation:

*What shocked me*, *however*, *was the interconnectedness between all pieces of evidence which I guess simply corresponds to the nature of medicine.* (P16 R1)

And thirdly, preparing for the second meeting at the end of the first-year participants were rewarded with a sense of achievement:

*As I reflected on my progress*, *I was actually amazed and proud of the amount of content that I had learned.* (P16, R2)

### Discovery through dialogue

Participants discovered that portfolio meetings were less anxiety-provoking and more helpful than anticipated. Although they described feeling nervous before their first meeting, in both the interviews and written reflections, this was resolved when [the meeting] “*was significantly more relaxed than…expected*.” (P42, R1) This enhanced a readiness to share and learn– one of the preconditions for TL (Mezirow, 1991). After the first meeting discoveries included an affirmed sense of achievement. After the second meeting participants had discovered new goals and strategies.

Four helpful discoveries from the meeting dialogue with the Advisor were described:

#### Progress

Many participants described feeling reassured about their progress after the first and second meetings, especially when Advisors were more positive than their own impressions from preparing for the meeting. For example, P14 found [the meeting] “*changed my perspective toward my progress because it is a lot better than my expectation*”. (P14, R1)

The second meeting also provided reassurance and confidence about progress.

*It was affirming to hear that my Advisor believed I had progressed to the same degree (or beyond) the progress I felt I had made…* (P31, R2).

#### Areas for improvement

Beyond a general sense of progress, the meetings provided new insights into areas of success, areas for improvement, and the effectiveness of study approaches:

[the discussion] *made me a bit more honest with myself*, *made me realize inefficiencies in my methods and ways I can improve.* P21 R1.

#### Strategies and goal setting

Clarity about areas of success or otherwise led to participants being more receptive to advice about strategies and goal setting. Many described new study and self-care strategies after the first meeting. For example:

*The interview conducted had helped not only by providing alternate ways to learn better but also improve my lifestyle holistically. My interviewer had given me tips on better self-care*, *how to increase productivity during the ongoing Covid-19 pandemic and ways to improve my study habits.* P40 R1.

Some described adapting their approach to goal setting after the first portfolio meeting:

*The last learning plan had perhaps too much of an emphasis on results rather than the process of learning*, *so I have tried to adjust my learning plan to focus more on strategies.* P13, R1.

After the second meeting, many reported a new awareness of how goal setting could be helpful. For example:

*It forced me to actually come up with a plan rather than having a blasé attitude towards my studies. They also brought a sense of achievement when I later achieved the goals that I had written about.* P37 R2.

Participants also realized the importance of setting the right goals:

*I needed to start asking myself what I really want to achieve and set goals I really want to meet.* P11 R2.

*I was able to visualize my goals and my weaknesses further to provide a purpose in my learning towards becoming a medical practitioner.* P7 R2.

Not only were goals revised and valued through portfolio meetings, but participants also expressed renewed motivation because of goal commitment:

*In verbalizing my learning goals and making a commitment to achieve them*, *my motivation to do so was much greater. P8 R1*.

#### A new purpose

The greatest change of heart described by participants during interviews was a new understanding of the purpose of studying medicine.

*So in high school*, *… you’ve got to get these marks*, *and to get these marks you have to study. To study you have to learn all of this content. So*, *it was purely academic… Then*, *it was only as I started to look into the interview…* [there was the realization] *that medicine is not so narrow as exams and traditional academia*, *…but most importantly*, [it is about being] *good enough to be a doctor in the future.* (P7, T1)

In the written reflections, participants described a newfound appreciation for the value of portfolios. This was elaborated in the interviews.

*I think when I just spoke to you the first time*, *I was like*, *“Man*, *what’s the point of this? It’s just a waste of time.”… it’s to give us the opportunity to pause and reflect on what is working*, *what’s not working.* (P3, T2)

### Dialogue disappointment

Some participants, however, remained doubtful of the benefits of portfolio meetings. P1, who had initial reservations about the disclosure or self-presentation, remained circumspect after the second meeting. After the second portfolio meeting, P1 acknowledged that any benefits from the meetings may have been limited by a lack of disclosure:

*Maybe that’s my fault. I don’t go in there with anything very challenging. I don’t take the opportunity of portfolio interviews* [meetings] *to raise concerns about the course or complaints about anything.* (P1, T2)

When meetings did not bring discoveries about progress, goal setting or purpose, misconceptions about the value of portfolios persisted.

P1 explained that both meetings were unhelpful because P1 *“had done all the talking”*, meaning there was no time for the Advisor to provide feedback or advice.

*As an opportunity to get feedback on your learning progression*, *they* [meetings] *haven’t worked for me yet.* (P1, R2).

P20, in a written reflection, described the second portfolio meeting as “*too rushed*” and that the advice received was unhelpful:

*Academically*, *I was told to not prioritize marks and rather my health*, *as I had been demonstrating unhealthy patterns of studying behavior. I was told to focus toward more sustainable methods of study. However*, *I didn’t feel this was the issue; rather*, *my discipline was more of an issue.* (P20, R2).

In this instance, the meetings did not change the participants’ perspective or behavior.

In summary, between the first and second meetings, many participants described changes in their perspectives on the curriculum, their progress, the benefits of reflection and portfolios and their approach to goal setting and study planning.

In the final stage of analysis, we used TL theory(Mezirow, 1991; Taylor, 2012) to interpret how our themes were connected to explain changing participant’ perceptions over time. The two themes representing preconceptions or initial perceptions (*Disclosure Dilemma and the Question of Priorities*) influenced the choices of experiences represented in the portfolio presentations. If new insights about the curriculum and personal progress occurred during preparation for the meeting (*Seeing the big picture*) or during portfolio meetings (*Discovery through Dialogue*), these preconceptions were transformed. This in turn enhanced the students’ readiness to share and learn in the second meeting, enabling a virtuous cycle of discovery through meeting preparation, honest disclosure, and critical reflection. Meetings that disappointed perpetuated doubts and reinforced preconceptions. This limited the priority participants gave to preparation, honest disclosure, and readiness for discovery. This inter-relationship between preconceptions, experience and transformative learning is explained in TL theory. (Mezirow, 1991; Taylor, 2012)

A visual display was constructed by JM and refined by the other members of the research team through discussion and consensus to illustrate how our themes, the portfolio tasks (preparation and meetings), timing (before, during or after meetings) and the concepts of TL theory (preconceptions, readiness, critical reflection, and perspective transformation) were interconnected.

## Discussion

Our findings provide evidence that preparation for and discussion during portfolio meetings can support new perspectives on learning, reflection, portfolios and approaches to study planning. However, student preconceptions represented by the themes of *Disclosure Dilemma* and *A Question of Priorities* created an initial barrier to TL and the development of new perspectives during the first meeting. When these barriers were overcome reflection on meaningful experiences during preparation (the theme *Seeing the Big Picture)* provided a more nuanced understanding of progress, including strengths and areas for improvement, the relevance of the curriculum and the value of reflection. Recognizing what had been learned, what needed to be learned, and how and why, created conditions for a student-focused dialogue about study purpose, goal setting and new strategies for learning. That meant insight gained during meeting preparation supported TL during portfolio meetings. Conditions became more favorable for TL if the first meeting was perceived as helpful. When time spent preparing for meetings and honest disclosure was rewarded, a virtuous cycle of disclosure and discovery occurred. Readiness to share and learn, one of the recognized preconditions for TL (Taylor, 2012), allowed problematic preconceptions to be resolved.

Concerns about disclosure created a dilemma for participants about what to share with an unknown academic in an unfamiliar context. Participants needed to balance the risk of losing face by acknowledging difficulties, with the risk of not receiving personalized advice (Cornally & McCarthy, 2011). This dilemma may be compounded by the prospect of assessment, which creates pressure for positive self-presentation (Heeneman & de Grave, 2017; Loosveld et al., 2023; Meeuwissen et al., 2019; van der Gulden et al., 2022; Watling & Ginsburg, 2019). A lack of honesty risks perfunctory reflections (Driessen et al., 2005) and irrelevant or fragmentary evidence that provides little insight into progress or areas for improvement (Oudkerk Pool et al., 2020). This is problematic because a shared understanding of the student’s strengths and areas for improvement is critical to meaningful dialogue during portfolio meetings about goal setting (Wolff et al., 2018).

Portfolio task design can scaffold the evidence students present during meetings with mentors (Van Tartwijk & Driessen, 2009). However, for students to present both successes and difficulties, they need to trust the process and their mentor (Arntfield et al., 2016; Daloz, 2012; Hauer et al., 2022). Trust is also needed before students can accept new information that challenges their own beliefs (Taylor, 2007; Telio et al., 2015). Trust can be fostered when mentors are supported and trained in meeting management, reflective dialogue and relational skills (Wolff et al., 2021). It can also be helpful when mentors share their own vulnerabilities (Arntfield et al., 2016) and if the purpose of the portfolio meeting is clarified early (Beckers et al., 2016; Loosveld et al., 2023). Consequently, for portfolio meetings to elicit honest exchange for TL, an “educational alliance” between students and mentors with shared purpose, goals, respect, and trust is needed (Telio et al., 2015). In this study, many participants described being less anxious before their second portfolio meeting because they understood what was needed and trusted their Advisor to be receptive and supportive. When the initial meeting was perceived to be helpful, students were more likely to have positive expectations for subsequent meetings.

Another condition that promoted engagement in portfolio meetings was an appreciation for reflection on learning that arose during the required preparation for the first meeting. This helped to offset misconceptions about portfolios and portfolio tasks represented by the theme *A Question of Priorities*. In our study setting, portfolio meetings are compulsory but are not the basis for decisions about progression. This may alleviate the types of tensions found where portfolios have a combined role in programmatic assessment and support (Heeneman & de Grave, 2017). However, some of our participants may have prioritized knowledge-based assessments over portfolio preparation, limiting their readiness to engage in discussions and consider alternative perspectives about study strategies and goals offered by their Advisor. This may explain why some participants described no change in their perspectives or study approaches after portfolio meetings in our study and why mixed student perceptions about the benefits of portfolios have been reported in previous research (Buckley et al., 2009; Tan et al., 2022; van der Gulden et al., 2022).

It was only after the second portfolio meeting, when conditions for TL were more favorable, that many participants described a changed perspective on the benefits of goal setting in both their interviews and written reflections. Goal setting is important for learning because it enhances student autonomy and motivation (ten Cate et al., 2012). Realistic goals, contingent on a clear understanding of progress and the purpose of learning, direct new activity (Friedman, 2022). An added benefit is that the negotiation and co-construction of goals strengthen an educational alliance (Farrell et al., 2019). For some participants, however, in the case of *Dialogue Disappointment*, negative preconceptions persisted because TL had not occurred. Portfolio meetings perceived as unhelpful created a vicious cycle underscored by doubt about the value of portfolios. Without readiness to share and learn in portfolio meetings, TL cannot occur.

Our findings suggest that initial experiences shape later students’ perceptions of portfolio tasks and meetings. This reinforces the importance of mentor, coach or advisor selection, training, support, and supervision of their communication, meeting management and coaching skills (Loosveld et al., 2022; Meeuwissen et al., 2019; Sheri et al., 2019; Wolff et al., 2021). Equally, students need training and support to identify and resolve sources of misapprehension before and after meetings. Portfolio tasks, including the curation of experiences for discussion in meetings, should be purposefully designed to scaffold the presentation of experiences that are meaningful to the student’s learning, representative of the curriculum, and include challenges. For example, in guiding reflection for TL students could be asked to consider the relevancy of the experience to future practice, and the next step on the path to competency. This would frame reflection towards the purpose for learning and an area for improvement. Such an approach would depend on the mentor’s understanding of the student’s motivation for learning. Our findings suggest that the conditions for reflective dialogue and transformational learning depends on skillful management of student misconceptions, mentor training and students’ willingness to reveal meaningful experiences that honestly represent their challenges and progress toward clinical competency.

### Strengths and limitations

Despite being limited to one cohort of students in one medical school setting, our study design prospectively documented how the perceptions of a diverse sample of students changed during the first years of medical education. Our findings were strengthened by two types of data collected by different means and for different purposes but that were both directly relevant to student portfolio experiences. Our theoretical lens revealed potentially transferable findings on how meeting preparation and dialogue support TL, adding to extant portfolio research on how portfolios promote reflection skills and SRL.

The written reflections, prepared as an assessment task for the portfolio meetings, were likely skewed toward a positive view of portfolio meetings; thus, it was necessary to look for and examine alternative views using confidential interviews. The descriptive quality of the written reflections also limited the depth of analysis possible with this source of data. We acknowledge that the participants who volunteered for the interviews were likely to have had stronger views about portfolio learning, and their perceptions of the portfolio meetings were shaped through repeated reflection during interviews with the research assistant. The sample attrition for the interview data may have provided an additional bias limiting our interpretation of the influence of time and experience.

The study captured participants’ perceptions of their first two portfolio meetings in first year. Any positive shifts in perceptions or doubts expressed may not have continued after the study period. The period also included the shift to online learning during the coronavirus pandemic, which impacted the participants’ learning in the medical program. During lockdowns, the opportunity to reflect and meet individually with academic staff may have been more strongly valued than if they were learning through face-to-face activities, with peers, teachers, and patients.

### Further research

Further research is needed to explore how students respond to portfolio meetings in other educational contexts and portfolio designs, including programs where portfolios incorporate programmatic assessment. Better understanding of conditions such as aspects of portfolio design and delivery including meeting frequency and format, mentor training and student preparation on transformation outcomes is also needed. Ultimately, it would be valuable to study whether early experiences and skills learned through portfolio meetings in preclinical years are transferred to clinical contexts and thus prepare students for life-long learning as practicing doctors.

## Conclusion

Portfolio meetings have the potential to serve as spaces for transformative learning; when students are well prepared, trust is established, and reflective dialogue occurs based on honest exchange. In our study, transformative learning was apparent when portfolio meetings led to new insights into portfolio tasks, learning progress, the curriculum, study strategies and goal setting. Early misapprehensions, including fears of self-disclosure and doubts about the benefits of portfolios, could be dispelled by experiences of supportive and helpful first portfolio meetings. Preparatory reflection on a collection of artifacts enhanced readiness to engage in the portfolio meetings and an appreciation for reflection. Our findings demonstrate the importance of scaffolded portfolio collections to support reflection, student preparation that anticipates misapprehensions, and mentor training to ensure safety for student disclosure about challenges and successes during the transition to medical studies. We suggest further research in other contexts and to explore whether early portfolio meetings shape later engagement with portfolio-based learning in clinical contexts.

## Electronic supplementary material

Below is the link to the electronic supplementary material.

Supplementary Material 1

## Data Availability

No datasets were generated or analysed during the current study.
