# Supplementary material for: Discoveries or doubts: a qualitative study of the transformative potential of portfolio meetings
Source: Adv Health Sci Educ Theory Pract. 2024 Oct 31;30(4):1045–64. doi: 10.1007/s10459-024-10387-3 (PMC12390870; doi:10.1007/s10459-024-10387-3)
Supplement: Supplementary file 1 — Supplementary Material 1 [file 10459_2024_10387_MOESM1_ESM.pdf]

Interview 1 schedule

Can you tell me about how you came to study medicine at Western Sydney, from when you first started to consider studying medicine to when you found out you were accepted into medicine? [Icebreaker question to provide some understanding of student and to settle them in. No prompts but used to lead into next question]

How do you plan your study?

Can you give me an example?

How do you keep track of your learning?

For example, have you ever kept a portfolio, journal, or something similar to track your learning?

Describe how that worked for you…

What benefits can you imagine? Any disadvantages?

What are you first impressions about the portfolio?

Was there anything surprising you found out about yourself in preparing for the first interview?

It's difficult to assess your own level of knowledge and skills...

Can you describe a time when you got it wrong? That is, when you *over or underestimated* your abilities or depth of knowledge.

What happened?

Why does that event stand out?

Do you have any theories for why that happened?

How did the experience affect you?

Is there are moment or memory when you judged your knowledge or skills accurately? Tell me the story.

How did you know you got it right?

What did you learn about yourself from that experience?

We would like to interview you again in a year to see how you are going. Would you be OK if we contacted you again? You can decide whether to participate then. Yes/no

Interview 2 schedule

Let's start by talking how the last year has been for you.

Tell me about your first year of studying in medicine – starting from the orientation week up until now.

Can you describe an experience at uni that has made a significant impact on your study?

What did you learn about yourself from that experience?

Any other notable experiences?

Is there an achievement in the past year that you are particularly proud of? Tell me about that…

Has there been anything that has been challenging for you in the past year? Can you describe that for me?

What impact did/does that have on you?

What about outside uni - have there been any notable experiences for you in the past year? How was it important to you?

Now about the portfolio

Could you describe for me how you have used the portfolio since the first workshop?

> Describe how and when you collect evidence… Can you give me some examples?
>
> What are the things you consider when you create your showcase?

What sort of thing would you avoid choosing? Why?

Tell me your thoughts when you looked at your completed showcase?

What were your thoughts about your achievements over the year?

What do you notice about yourself in your choices?

Tell me about your interviews with the advisors?

Can you describe what happened during the interview? What was it like for you?

How did you prepare for the interview?

How was the first interview different to the second? Can you explain?

Can you describe the feedback you received?

What was the impact of the feedback? How did you feel? Did it lead to any changes?

Describe how your ability to assess your knowledge or skills has changed since starting med school?

Is there are a moment or memory in the past year when you judged your knowledge or skills accurately?

Tell me the story.

How did you know you got it right/wrong?

And can you describe a time when you got it wrong/right?

Why does that event stand out?

How did that experience affect you?

Is there anything else you would like to share with me?

Interview 3 schedule

I understand how difficult Covid has been for your studies.

What's top of your mind about you study for you now?

I remember last year you talked about some significant learning experiences. Have there been any stand-out experiences this year? Can you tell me about it?

Was there anything about that experience that you found challenging or gave you a sense of achievement?

Who has been an important influence on your learning this year?

Tell me about a time when this person or these people influenced your learning…

How has your approach to assessing your progress changed since the last time we spoke? Tell me…

What are the most important influences?

Can you tell me a story about that?

Many students told us in the first interviews was they sometimes purposefully underestimated their skills

Can you give me an example from this year if you have done this?

Now about the portfolio…

Have you learnt anything about new about yourself in your choices for the portfolio showcase this year?

Can you explain by giving an example?

After the interviews last week can you describe how you felt about your progress so far in medicine?

And finally, in the first interviews some students described a sense of not yet belonging in medical school.

Can you tell me about a recent time when you felt like you belonged in medical school?

What about a time when you didn't belong?

How did that change how you feel about your future?

Is there anything else you would like to share with me about the portfolio or self-assessment?

Portfolio meeting written reflection guidelines

Describe and evaluate the portfolio meeting.

What did you learn from the preparation for the portfolio meeting and the experience of the meeting?

Did this change your view of your progress?

Have you made any changes to your learning plan since your portfolio meeting?
